# Left bundle branch area pacing vs right ventricular pacing in preserved or mildly reduced ejection fraction: the PACE-HF trial

**DOI:** 10.1093/eurheartj/ehaf724

**Published:** 2025-10-15

**Authors:** Jenish P Shroff, Deep Chandh Raja, Sreevilasam P Abhilash, Abhinav Mehta, Walter P Abhayaratna, Pugazhendhi Vijayaraman, Prashanthan Sanders, Rajeev K Pathak

**Affiliations:** The School of Medicine and Psychology, Australian National University, Canberra, ACT, Australia; Canberra Heart Rhythm Centre, Garran, Canberra, ACT, Australia; Canberra Heart Rhythm Centre, Garran, Canberra, ACT, Australia; Canberra Heart Rhythm Centre, Garran, Canberra, ACT, Australia; The School of Medicine and Psychology, Australian National University, Canberra, ACT, Australia; The School of Medicine and Psychology, Australian National University, Canberra, ACT, Australia; Geisinger Heart Institute, Geisinger Commonwealth School of Medicine, Wilkes-Barre, PA, USA; Centre for Heart Rhythm Disorders, University of Adelaide and Royal Adelaide Hospital, Adelaide, Australia; Canberra Heart Rhythm Centre, Garran, Canberra, ACT, Australia

**Keywords:** Left bundle branch area pacing, Right ventricular pacing, Heart failure

## Introduction

Chronic right ventricular pacing (RVP) may result in heart failure (HF).^1^ Advances in pacing strategies, including cardiac resynchronization therapy and, more recently, left bundle branch area pacing (LBBAP), aim to mitigate this risk.^2,3^ However, evidence supporting LBBAP over RVP in patients with preserved or mildly reduced left ventricular ejection fraction (LVEF) remains largely observational.^4^

## Methods

**Figure 1:**
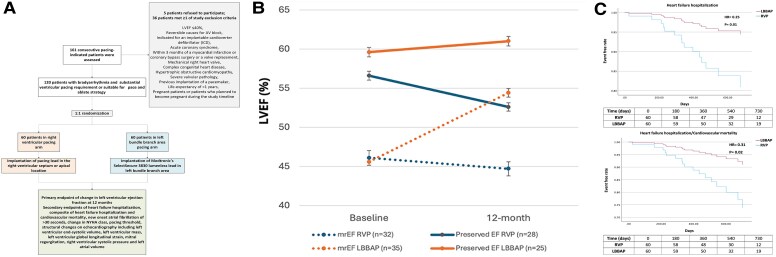
(*A*) CONSORT flow diagram of the randomized trial. (*B*) *Post hoc* analysis of change in left ventricular ejection fraction in subgroups defined by baseline left ventricular function. Among patients with mid-range left ventricular ejection fraction (41%–50%), left bundle branch area pacing (*n* = 35) resulted in significant improvement from 45.6 ± 3.1% to 54.4 ± 6.6%, while right ventricular pacing (*n* = 32) showed a decline from 46.1 ± 3.4% to 44.7 ± 6.7% (change in left ventricular ejection fraction: +8.8 ± 8.0% vs −1.5 ± 6.0%; *P* < .001). In those with preserved left ventricular ejection fraction (>50%), right ventricular pacing (*n* = 28) showed a reduction from 56.6 ± 3.2% to 52.6 ± 6.5%, while left bundle branch area pacing (*n* = 25) maintained left ventricular systolic function (59.6 ± 5.5% to 61.0 ± 5.4%; change in left ventricular ejection fraction: −4.0 ± 6.2% vs +1.4 ± 5.6%; *P* < .001). (*C*) Kaplan–Meier survival curves for heart failure hospitalization (HFH) and the composite of heart failure hospitalization or cardiovascular mortality comparing right ventricular pacing vs left bundle branch area pacing

This prospective, randomized, unblinded trial enrolled 120 patients aged ≥18 years with LVEF > 40% and an indication for permanent pacing due to atrioventricular block, tachy-brady syndrome with anticipated ventricular pacing > 40%, or permanent atrial fibrillation (AF) undergoing a ‘pace and ablate’ strategy. Patients were randomized 1:1 to RVP or LBBAP (*Figure 1A*). The study received ACT human research ethics committee approval, conformed to the Declaration of Helsinki, and was registered with ANZCTR (ACTRN12624001420538).

### Pacing procedure

In the RVP arm, a stylet-driven lead was positioned on the mid-apical interventricular septum. The LBBAP was performed using the Medtronic 3830 lead as previously described.^5^ Deep septal pacing (DSP) was accepted in absence of left bundle branch pacing (LBBP) or left ventricular septal pacing (LVSP).

### Procedural data

Lead implant time and fluoroscopy dose/time were recorded. V6RWPT, V6–V1 interpeak interval, and QRSd were measured using Labsystem Pro™ as previously described.^5^ Post-implant electrocardiograms of RVP arm and follow-up electrocardiograms of LBBAP arm were digitally analysed using e-Scribe™. Achievement of LBBP, LVSP, DSP, or microdislodgement on follow-up, defined as loss of R′ in V1 and >10 ms prolongation in V6-R wave peak time,^6^ was adjudicated by an independent cardiologist.

### Study endpoints

The primary endpoint was change in LVEF at 12 months, assessed by a blinded, independent operator in an accredited core lab using Simpson biplane method. Two readings were averaged; a third was obtained if variability exceeded 5%. Intra-observer reliability, assessed in 40 randomly selected samples, was excellent (intra-class correlation coefficient 0.92). Secondary endpoints included heart failure hospitalization (HFH), composite of HFH and cardiovascular death, new-onset AF > 30 s, NYHA class, pacing threshold, left ventricular end-systolic volume (LVESV), left ventricular (LV) mass, global longitudinal strain (GLS), right ventricular systolic pressure (RVSP), mitral regurgitation (MR), and left atrial (LA) volume. Heart failure hospitalization was defined as any presentation requiring intravenous diuretics, including emergency visits and short-stay admissions (<24 h).

### Statistical analysis

A sample size of 60 per group was calculated to detect a 5% difference in LVEF assuming a standard deviation of 8%, with 90% power, *α* = .05, and 10% dropout margin. Continuous variables are reported as mean ± SD and compared using *t*-tests. Categorical data were compared using *χ*² or Fisher’s exact test. Multivariable linear regression with stepwise selection was used to identify independent predictors of LVEF change. Time-to-event endpoints were analysed using Kaplan–Meier curves and log-rank tests. Statistical significance was defined as *P* < .05. Analyses were performed using SPSS v29 (IBM, NY, USA).

## Results

### Baseline and procedural characteristics

The groups were well balanced in age, sex, pacing indication, comorbidities, medication use, NYHA class, baseline QRS morphology/duration, and echocardiographic parameters including LVEF (∼51%). However, implant duration [4 (3–5.3) vs 11 (7–16.5) minutes], fluoroscopy dose [14 (9.9–30.4) vs 41 (22.5–68.5) mGy], and fluoroscopy time [3 (2–4.9) vs 7.3 (5.6–10.9) minutes] were significantly shorter in the RVP group (all *P* < .001). Pacing thresholds at implant were low and similar. In the LBBAP group, LBBP was confirmed in 85%, with LVSP and DSP in 8% and 7%, respectively. Paced QRSd was narrower with LBBAP (134 ± 17 vs 161 ± 20 ms; *P* < .001). One RVP patient required wound re-exploration; no device-related complications occurred in the LBBAP arm.

### Primary endpoint

Left ventricular ejection fraction improved significantly in the LBBAP group at 12 months compared with RVP (57 ± 7% vs 48 ± 8%; *P* < .001). The ΔLVEF was 5.7 ± 7.8% in LBBAP vs −2.7 ± 6.3% in RVP (*P* < .001), despite a higher pacing burden in LBBAP (81 ± 24% vs 70 ± 26%; *P* < .001). In multivariable analysis, LBBAP independently predicted higher LVEF (adjusted mean difference: +8.1%; 95% CI: 5.6–10.6; *P* < .001).

### Subgroup analysis

In patients with mid-range LVEF (41%–50%), LBBAP significantly enhanced LVEF (8.8 ± 7.8%, *n* = 35), whereas RVP was associated a decline (−1.5 ± 6.4%, *n* = 32, *P* < .001). Pacing burden in these subgroups mirrored the parent cohort (RVP: 69 ± 26% vs LBBAP: 83 ± 25%; *P* = .02). In preserved LVEF, RVP (*n* = 28) was associated with a reduction in LVEF (−4.0 ± 6.2%) vs minimal change with LBBAP (*n* = 25, 1.4 ± 5.6%; *P* < .001; *Figure 1B*). A ≥10% LVEF drop occurred in nine RVP patients vs one in LBBAP, who had received LVSP instead of LBBP.

### Secondary endpoints

Heart failure hospitalization occurred more frequently in RVP than LBBAP (20 vs 5%, *P* = .01) as was the composite of HFH and cardiovascular death (22 vs 6.7%, *P* = .02). New-onset AF (>30 s) was more common with RVP (14.3% vs 2.1%; *P* = .03); three RVP patients had AF exceeding 24 h whereas the lone LBBAP patient had AF of <30 min. At 12 months, 85% of LBBAP patients were NYHA I vs 58% with RVP; NYHA III/IV persisted in 12% of RVP and none of LBBAP. Adjusted analysis confirmed significantly better NYHA class in the LBBAP arm (mean difference: −0.44; 95% CI: −0.63 to −0.26; *P* < .001). Left bundle branch area pacing led to reductions in LVESV (−10.5 ± 18.9 vs 2.7 ± 18 mL; *P* < .001), LV mass (−5.7 ± 16 vs 4 ± 11 g/m²; *P* < .001), and improved LV GLS (−1.2 ± 3 vs 2.7 ± 5.7%; *P* < .001). The ΔLA volume was not significantly different (−2.4 vs + 5.7 ml; *P* = .07). Right ventricular systolic pressure remained unchanged in both groups. New/worsened MR was numerically higher in RVP (10% vs 1.7%, *P* = .054). Pacing thresholds remained stable at 12 months (both 0.9 ± 0.6 V); one patient per group had a >1 V rise. Loss of LBB capture occurred in three patients (6%). No lead revision was required in either arm.

## Discussion

Right ventricular pacing was associated with lower adjusted LVEF at 12 months compared with LBBAP (−8.5%; 95% CI: −10.85 to −6.23; *P* < .001), aligning with STAY trial results.^7^ Although increased pacing is typically detrimental, the physiologic synchrony achieved with LBBAP may render higher pacing burden beneficial in patients with AV block and LV dysfunction. Extending our prior findings among patients with LVEF ≤ 35%,^8^ LBBAP also improved outcomes in patients with LVEF > 40% with higher HFH-free survival (95% vs 80%; *P* = .01) and combined HFH or cardiovascular mortality-free survival (93.3% vs 78.3%; *P* = .02; *Figure 1C*).

### Limitations

This single-centre study with a limited sample size reduces generalizability; validation in ongoing multicentre trials (e.g. PROTECT-HF and PROTECT-SYNC) is warranted. The high pacing burden observed may restrict applicability to patients with lower ventricular pacing needs; nonetheless, the findings support the use of advanced pacing strategies to minimize RVP.^9^ Most participants had narrow QRS, limiting relevance to those with wide QRS or distal conduction disease, where comparison with biventricular pacing may be more appropriate. Atrial fibrillation detection relied on device diagnostics, which carries less prognostic weight than clinically manifest AF.^10^ Finally, subgroup analysis was *post hoc* and should be considered exploratory.

## Conclusions

Left bundle branch area pacing was superior to RVP in preserving LVEF and reducing HFH and cardiovascular mortality in patients with preserved or mildly reduced LVEF requiring substantial ventricular pacing.

## Declarations

### Data Availability

The data that support the findings of this study are available from the corresponding author upon reasonable request.

### Pre-registered Clinical Trial Number

The trial was registered with ANZCTR, and the clinical trial number is ACTRN12624001420538.
